# Copy of Dr. Redwood’s Report on Trommer’s Extract of Malt

**Published:** 1878-11-20

**Authors:** T. Redwood

**Affiliations:** Professor of Chemistry and Pharmacy to the Pharmaceutical Society of Great Britain; 17, Bloomsburg Squabe, London


### COPY OF DR. REDWOOD'S REPORT ON TROMMER'S EXTRACT OF MALT.

17, BLOOMSBURG SQUARE,

LONDON, Sept. 18, 1878.

*Dr. Redwood's Analytical Department*:

I have examined the Extract of Malt manufactured by the "Trommer Extract of Malt Company," and judging from its physical characters and chemical reactions, I am of opinion that it fairly represents what its name indicates, that is, that it is a preparation of Malt, in which are contained the essential properties of that substance, with a slight addition of Aromatic Bitter of the Hop. It has the character of a soft Extract, in the sense in which that term is used pharmaceutically, and it has evidently been prepared with great care and judgement, as it retains the property of acting on amylaceous bodies, as diastase does, while the Extract itself bears long keeping without change.

It also possesses the property of forming with Cod Liver Oil, a permanent Mixture or Emulsion, in which the taste of the Oil is very effectually covered, and its administration thus greatly facilitated. T. REDWOOD, Ph. D., F. R. C. S., &c.

Professor of Chemistry and Pharmacy to the Pharmaceutical Society of Great Britain.

1t

☞ Friends, the year draws to a close—Let all who are in arrears for subscription, remit the amount at once.

